# Clinical Outcomes of Patients with Chronic Myeloid Leukemia and COVID-19 Infection—A Single Center Survey

**DOI:** 10.3390/medicina59091564

**Published:** 2023-08-28

**Authors:** Irena Ćojbašić, Ivana Golubović, Žarko Ćojbašić

**Affiliations:** 1 Faculty of Medicine, University of Niš, Blvd. Zorana Đinđića 81, 18000 Niš, Serbia; icojbasic@gmail.com; 2 Clinic of Hematology, Allergology and Clinical Immunology, University Clinical Centre Niš, Blvd. Zorana Đinđića 48, 18000 Niš, Serbia; ivanica12984@gmail.com; 3 Faculty of Mechanical Engineering, University of Niš, Aleksandra Medvedeva 14, 18000 Niš, Serbia

**Keywords:** chronic myeloid leukemia, COVID-19, tyrosine kinase inhibitors

## Abstract

*Background:* Previous research has shown different effects of hematological malignancies on the outcome of patients with COVID-19 infection depending on the type of disease and the treatment received. This research was aimed at examining the clinical outcome of COVID-19 infection in positive patients with chronic myeloid leukemia treated with tyrosine kinase inhibitors. *Methods:* We collected retrospective information on chronic myeloid leukemia patients who were treated and monitored in our institution during the pandemic period. Within this cohort, we recorded COVID-19 positive symptomatic patients and analyzed their basic characteristics, symptoms, severity, and outcome. *Results*: In the study cohort when COVID-19 was diagnosed, 86.7% of patients were on first-generation tyrosine kinase inhibitors therapy—imatinib. At the time of infection, 70% of patients were in molecular remission, 23.4% in complete cytogenetic remission, and 3.3% in complete hematological response. Most patients had symptomatic disease. Within the analyzed group, 56.7% of patients had asymptomatic/mild COVID-19 infection, 23.3% of patients had moderate symptoms which did not require hospitalization, and 20% of patients had severe/critical symptoms that required admission to the intensive care unit. More than half of the patients interrupted treatment with tyrosine kinase inhibitors temporarily during COVID-19. There were no deaths due to COVID-19 infection. *Conclusions*: In compliance with other larger clinical studies, analysis of the clinical outcome of COVID-19 infection in patients with chronic myeloid leukemia on tyrosine kinase inhibitors therapy in this study showed that they do not have an increased risk for COVID-19 infection and that they have a mild course of the disease with recovery.

## 1. Introduction

Chronic myeloid leukemia (CML) is a malignant hemopathy resulting from reciprocal translocation of ABL1 from chromosome 9 and BCR from chromosome 22 leading to the formation of a hybrid BCR::ABL gene from which a new BCR::ABL protein is derived, that plays a central role in the development of chronic myeloid leukemia [1]. Great progress in the treatment of patients with chronic myeloid leukemia has been made with the introduction of tyrosine kinase inhibitors (TKIs), which is the first targeted molecular therapy since it is a competitive inhibitor of the BCR::ABL tyrosine kinase protein. Several TKIs have been approved for the treatment of chronic myeloid leukemia, and the choice of therapy for individual patients is determined by considering the efficacy, tolerability, and cost of therapy [2].

Coronavirus disease 2019 (COVID-19) is an infectious disease caused by the newly discovered coronavirus SARS-CoV-2, which has a wide range of clinical presentations, from asymptomatic infection to serious disease requiring treatment in the intensive care unit and respiratory support [3]. Most patients have mild to moderate respiratory disease, but the clinical course can be severe in elderly patients as well as in patients with comorbidities including hematological malignancies [4,5].

Persons with hematological cancers have a similar case rate of COVID-19 infections compared to the general populations but have more severe disease with higher rate of complications and mortality in relation to them [5].

The mortality of patients with hematological malignancies and COVID-19 was nearly four times higher than that of the general population with COVID-19 infection. The mortality of patients with hematological malignancies and COVID-19 was 41 times higher than that of such patients without SARS-CoV-2 infection. Overall survival was independently predicted by age, type of malignancy, disease status, and the severity of COVID-19. It was found that older age, progressive disease status, diagnosis of acute myeloid leukemia, non-Hodgkin lymphomas, plasma cell neoplasms, and severe or critical COVID-19 were predictive for a poor outcome [4].

Based on the results of a systematic review and meta-analysis [6], the risk of mortality appears to be high and estimated at 34%, in hospitalized patients with hematological malignancies and COVID-19 infection. It is important to note that despite the worrying risk of death, most patients with hematological malignancies and COVID-19 infection survive and recover, even after recent administration of systemic anti-cancer therapy [6].

Although patients with chronic myeloid leukemia receiving tyrosine kinase therapy could potentially be at higher risk of developing COVID-19 infection than the general population, it has been shown that the absolute incidence rate is very low and clinical features are as in a healthy population [7]. The prevalence of infection in patients with chronic myeloid leukemia is similar to that in the general population, suggesting that they are able to elicit an appropriate response to antibodies against SARS-CoV-2. Most of the papers on chronic myeloid leukemia patients who had COVID-19 infection showed a mild course of the disease with recovery [8,9,10,11,12].

In the largest study to date [13], data were collected from 8665 patients with chronic myeloid leukemia, showing that the prevalence of COVID-19 infection was 2.5%. A third of the patients were aged between 50 and 65 years, while three quarters were men. More than half of the patients presented concomitant comorbidities. The largest number of patients, in total 78%, were only quarantined, while 9.6% required hospitalization without the need for respiratory assistance, and 8.2% were hospitalized for respiratory assistance. Only 15% of patients were not in molecular response at the time of the infection. In one quarter of patients, tyrosine kinase inhibitors therapy was discontinued during the infection. The mortality rate of COVID-19 was 5.5% in the positive cohort and 0.13% in the whole cohort.

This research was aimed at determining the clinical characteristics, severity, and outcomes of COVID-19 infection in positive patients with chronic myeloid leukemia.

## 2. Patients and Methods

### 2.1. Study Design and Study Population

A cohort of adult patients with chronic phase chronic myeloid leukemia who were receiving tyrosine kinase inhibitors therapy was analyzed. A series of 30 patients with chronic phase chronic myeloid leukemia who had COVID-19 infection during the 24-month period from March 2020 to February 2022 was identified. The diagnosis of COVID-19 infection was confirmed by a positive antigen or PCR test for SARS-CoV-2. Patient data were collected by retrospective analysis of medical records.

Data on the demographic characteristics and comorbidities of chronic myeloid leukemia patients who experienced COVID-19 infection, symptoms and signs of infection, severity of symptoms, applied therapy for COVID-19, and clinical outcome of the infection were analyzed. Data related to chronic myeloid leukemia, duration of the disease, type of tyrosine kinase inhibitor last used, last therapeutic response achieved, and whether the patient received tyrosine kinase inhibitor while COVID-19 positive were also analyzed. The main limitation of this study is the relatively moderate number of tyrosine kinase inhibitor-treated chronic myeloid leukemia patients that have been analyzed.

The latest recommendations of the European LeukemiaNet [2] were used for the diagnosis of chronic myeloid leukemia, determining the criteria for the chronic phase of the disease, monitoring patients during tyrosine kinase inhibitor therapy and criteria for therapeutic response. All patients started treatment with the recommended oral dose of imatinib of 400 mg once a day. In case of failure defined according to the current European LeukemiaNet criteria [2] or intolerance to imatinib, patients were switched to second-generation tyrosine kinase inhibitor nilotinib, at the dose of 800 mg daily. Nilotinib was the only second-generation tyrosine kinase inhibitor used in this study. The chronic myeloid leukemia risk scores were calculated according to the Sokal, Euro, and EUTOS formulations. To evaluate comorbidities, the globally used Charlson comorbidity index was applied, because the role in prognosis of stratification according to this score has been well established in patients treated with tyrosine kinase inhibitors.

Signs, symptoms, and severity of COVID-19 infection were assessed based on the World Health Organization’s (WHO’s) criteria from 2020 [14]: Asymptomatic—Individuals who are positive for SARS-CoV-2 but do not have COVID-19 compliant symptoms; Mild—Individuals who have any of the various signs and symptoms of COVID-19 (e.g., fever, cough, sore throat, malaise, headache, muscle aches, nausea, vomiting, diarrhea, loss of sense of taste and smell) but who do not possess shortness of breath, dyspnea, or abnormal chest image; Moderate—Individuals showing signs of lower respiratory tract disease during clinical evaluation or imaging and having SpO2 ≥ 94%; Severe—Individuals with SpO2 < 94%, arterial partial pressure oxygen ratio and inhaled oxygen fraction (PaO2/FiO2) < 300 mmHg, respiratory rate > 30 breaths/min, or pulmonary infiltrates > 50%; Critical—Persons with respiratory failure, septic shock, and/or multiple organ dysfunction.

The pandemic period has not changed the clinical management and patients in this study continued to perform regular visits as scheduled, with no obligation for routine screening for SARS-CoV-2 active infection prior to medical visits.

### 2.2. Statistical Analysis

A general mathematical and statistical package was used for statistical analysis. Data have been analyzed using Matlab^®^ software and its Statistics and Machine Learning Toolbox (MathWorks, Natick, MA, USA, version R2023a). Median and range, frequency, and percentages were used to summarize nonparametric quantitative data.

### 2.3. Ethical Approval

This study was appropriately approved by the local Ethics Committee of the University Clinical Center Niš, Serbia (date: 26 May 2022; number: 14396/3). The study was carried out fully following the Helsinki Declaration on Human Clinical Research.

## 3. Results

### 3.1. Basic Characteristics of COVID-19-Positive Patients with Chronic Myeloid Leukemia

Retrospectively, a total of 95 patients with chronic myeloid leukemia treated with tyrosine kinase inhibitors were analyzed. Thirty COVID-19-positive symptomatic patients during the 2-year pandemic were identified among the group, making 31.6% within this cohort. The characteristics of chronic myeloid leukemia patients who had COVID-19 infection are shown in Table 1. The mean age was 60.3 years (range 40–86 years), with 36.7% of patients being older than 65 years. Females were predominant with 56.7%. In the analyzed cohort, 43.3% were smokers and 26.7% of those were obese patients. The average time from diagnosis of chronic myeloid leukemia to COVID-19 infection was 8.4 years (range 6 months–18 years).

Comorbidities were found in 70% of patients at the time of infection and they received concomitant therapy. According to the Charlson comorbidity index, 36.7% of patients were classified as having a score of 1–2, 26.7% were stratified as having a score of 3–4, and only 6.6% of patients were classified as high risk with a score ≥ 5. The most common comorbidity was arterial hypertension with 50%, the second most common were cardiovascular diseases with 33.3%, and the third most common was diabetes mellitus, which was present in 23.3% of patients. The distribution of patients according to risk was similar according to the Sokal score (low 46.7%/intermediate 36.7%/high 16.6%) and the Euro score (low 53.3%/intermediate 36.7%/high 10%), while most of patients had a low EUTOS score, a total of 83.3%. At the time of diagnosis of COVID-19 infection, 86.7% of patients were on first-generation tyrosine kinase inhibitor therapy—imatinib, while only 13.3% of patients have been receiving second-generation tyrosine kinase inhibitor—nilotinib. The median time under tyrosine kinase inhibitor treatment was 7.8 years.

At the time of infection, the therapeutic response to tyrosine kinase inhibitor was a complete hematological response which had 3.3% of patients, a complete cytogenetic response was found in 20%, while 73.4% of patients had a confirmed molecular response (MR) in the range of MR3 (BCR/ABL1 ratio ≤ 0.1% International Scale (IS)) which achieved 13.4% of patients to deep MR (MR4, BCR/ABL1 ratio ≤ 0.01% IS, and MR4.5, BCR/ABL1 ratio ≤ 0.0032% IS) which was confirmed in 60% of the patients. One patient had no therapeutic response but was in a chronic phase of the disease. Thus, 26.6% of patients did not achieve a molecular response at the time of infection.

### 3.2. Symptoms, Severity, and Clinical Outcome in COVID-19-Positive Patients with Chronic Myeloid Leukemia

COVID-19 infection was confirmed by an antigen test in 17 patients (56.7%) or by a PCR test in 13 (43.3%) patients. The median time of swab positivity in the imatinib cohort was 10.4 days, while similarly in the nilotinib cohort it was 11.7 days. Of the total number of COVID-19 infected patients, 50% received the vaccine, where 36.7% received inactivated virus COVID-19 vaccines and only 13.3% received mRNA vaccines. The median time from last vaccination was 9.4 months. Of the vaccinated patients, 60% had COVID-19 infection prior to vaccination, while the remaining 40% were infected after the immunization, where no significant differences in the clinical course of the disease have been observed. The severity of infection, applied therapy, and the clinical outcome of patients with COVID-19 infection are shown in Table 2. At the time of diagnosis of COVID-19, the most common symptoms were fever which was present in 20 (66.7%) patients, followed by myalgia present in 16 (53.3%), and then cough present in 14 (46.7%) patients. Most of the patients, twenty-six (86.7%) had one or more symptoms of infection, while only four (13.3%) patients were asymptomatic.

According to the WHO criteria from 2020, asymptomatic and mild symptoms of COVID-19 infection were present in 17 (56.7%), moderate symptoms in 7 (23.3%), and severe/critical symptoms in 6 (20%) patients. Of the thirty COVID-19-positive patients, six patients (20%) required hospitalization in intensive care unit, where two patients had moderate pneumonia without hypoxia while four patients had severe pneumonia with hypoxia which demanded oxygen support. The rest of the patients, 24 of them (80%), recovered in isolation at home.

More than half of the patients, in total 16 (53.3%), discontinued tyrosine kinase inhibitor therapy while receiving specific therapy for COVID-19 infection, because of the potential drug–drug interaction (13 patients, 43.3%) or COVID-19 severity (3 patients, 10%). Patients identified the source of infection as being from a family member (40%), at work (26.7%), or being unknown (33.3%). All patients fully recovered after an average of 16 days of infection, range 5–38 days. There were no deaths due to COVID-19 infection.

## 4. Discussion

The treatment of patients with hematological malignancies, including chronic myeloid leukemia, was a challenge in the COVID-19 pandemic era [15]. Available data show a variable impact of different hematological malignancies on the outcome of the COVID-19 infection, taking into account the “protective” effect against the severe form of the disease due to a weakened immune response in some patients [6]. There are limited data published in the literature reporting on the outcome of treatment of patients with chronic myeloid leukemia and COVID-19 infection [7,11].

The overall incidence of COVID-19 infection in chronic myeloid leukemia is low compared to other hematological malignancies, which highlights the potential protective role of tyrosine kinase inhibitors. First-generation tyrosine kinase inhibitor—imatinib, with the inhibitory function of Abl1 which has been proven to affect the adequate performance of actin, which allows the virus to enter and fuse with the cell [16,17,18]. An in vitro study found that the protective function of imatinib takes place in the early stages of infection, since after internalization, the fusion of virions at the endosomal membrane is inhibited [9]. It was also shown that imatinib blocking the fusion could prevent the endocytosis necessary for viral activation of different viral species [11].

An in vivo study in mice showed that imatinib has the ability to reduce pulmonary edema, prevent cell damage, which may be a consequence of reduced release of proinflammatory cytokines such as IL-6, and tumor necrosis factor-alpha [19]. An analysis of 770 genes’ expression related to inflammation and immunity was carried out. Among the total number of analyzed genes, twenty were selected, where nine showed decreased expression during the administration of imatinib, which usually have a proviral function. Conversely, 11 of the selected genes had an increased expression as a result of the administration of imatinib, which could exert an “antiviral” role, by allowing the immune control of the host against the viral attack [18].

A recent study conducted on the chronic myeloid leukemia patients with COVID-19 infection determined a better clinical outcome of patients treated with imatinib, which is explained by the fact that a decrease in SARS-CoV-2/cell fusion occurred and the inhibitory activity of imatinib can interfere with the release or replication of the virus [20]. And finally, the results from real clinical practice show that patients with chronic myeloid leukemia and COVID-19 infection who were treated with imatinib have lower rates of admission to the intensive care unit, ventilatory mechanical support, and consequently also shorter hospitalization. Likewise, a twice-lower mortality rate was found in chronic myeloid leukemia patients receiving tyrosine kinase inhibitor compared to the control group [12].

The campus CML network collected retrospective information on 217 SARS-CoV-2-positive chronic myeloid leukemia patients followed across Italy during the first year of the pandemic [13]. Most positive patients, 66% of them, were under 65 years of age. The median time from diagnosis of chronic myeloid leukemia to COVID-19 infection was six years. In an Italian study, 56% of patients had concomitant comorbidities at the time of infection, of which arterial hypertension (34%) and diabetes mellitus (22%) were the most common. The analysis of this cohort determined the presence of comorbidities in as many as 70% of patients, while the most common comorbidities were arterial hypertension (50%) and other cardiovascular diseases (33.3%). Contrary to the results of an Italian study that showed that at the time of diagnosis of COVID-19 infection, 27% of the patients were receiving imatinib, and 52% of the patients were receiving second-generation tyrosine kinase inhibitor, in this study 86.7% were treated with imatinib and 13.3% were treated with nilotinib. The Italian campus CML network determined that at the time of infection, 85% of the patients achieved a molecular response in the range between MR3 and MR4.5, while that rate was lower in patients treated in our institution and amounted to 70%.

The CANDID study is a large global cohort study that analyzed the outcome of COVID-19 infection in 110 patients with chronic myeloid leukemia [21]. Chronic myeloid leukemia treatment at the time of COVID-19 diagnosis consisted of tyrosine kinase inhibitor in 70% of the patients, 36% were taking imatinib, 34% of patients were treated with second-generation tyrosine kinase inhibitor, while 16% of patients were untreated at the time of COVID-19 diagnosis for different reasons, mainly due to treatment-free remission. Asymptomatic patients made up 7%. In the group of symptomatic patients, COVID-19 infection was mild in 45% of patients, moderate in 17% of patients, severe in 17% of patients, and of unknown severity in 14% of cases. Similar results were obtained in this study, asymptomatic/mild disease made up 56.7%, moderate made up 23.3%, and severe/critical disease made up 20% of the patients.

In contrast to the CANDID study, where 30% of the patients reported discontinuation of tyrosine kinase inhibitor therapy during COVID-19 infection, this study showed a significantly higher discontinuation rate of tyrosine kinase inhibitor therapy of 53.3%. Of the total number of COVID-19 positive patients, in the CANDID study the outcome was favorable in 86% and fatal in 14%. In the cohort analyzed in this study there were no deaths due to COVID-19 infection. The CANDID study analyzed overall survival in patients with known outcomes by sex, age, comorbidities, duration of chronic myeloid leukemia, type of chronic myeloid leukemia therapy, discontinuation of tyrosine kinase inhibitor due to COVID-19, and administration of any treatment against COVID-19. Univariate analysis identified older age, COVID-19 severity, and imatinib treatment as predictors of mortality from COVID-19 infection [21]. Similar results were obtained by Italian researchers who found that older age, cardiovascular comorbidities, and imatinib therapy were associated with increased mortality from SARS-CoV-2 infection [13].

Treatment of chronic myeloid leukemia may present a unique problem during SARS-CoV-2 infection. Interactions between TKIs and drugs for the treatment of COVID-19 can be dangerous and require careful monitoring. Specific CML-related factors that affect the course of COVID-19 infection are also awaiting identification. There is growing evidence that tyrosine kinase inhibitor protects against COVID-19, however some implications of continuing tyrosine kinase inhibitor during COVID-19 are also concerning.

Therefore, the decision to discontinue or continue tyrosine kinase inhibitor during COVID-19 is difficult and requires careful consideration of several factors, including patient comorbidities and concomitant therapy, type of tyrosine kinase inhibitor, level of tyrosine kinase inhibitor response, previous adverse events, COVID-19 treatment, and severity of infection [10]. Results of the relevant studies [13,22] showed that in the group of patients who were in deep molecular remission at the time of COVID-19 infection and who continued treatment with tyrosine kinase inhibitor, none of the patients had to be hospitalized or needed treatment in the intensive care unit.

Tyrosine kinase inhibitors nowadays represent the golden standard in the treatment of patients with chronic myeloid leukemia. Although they are generally well tolerated, they may be associated with hematological and non-hematological side effects, which are usually not reasons for therapy discontinuation, unless an optimal response is lacking, or severe toxicity is registered. TKI-related hematological toxicity in the form of myelosuppression with consequent serious cytopenia may potentially increase the risk of severe COVID-19 infection. Non-hematological toxicity is common with all TKIs, and they can prolong the QTc interval, which can lead to torsade de pointes and sudden deaths [15,23].

Chloroquine and azithromycin were both used in the treatment of SARS-CoV-2 infection although there was no strong evidence that they were curative in patients with COVID-19. Since TKIs are metabolized via the cytochrome-P450, CYP3A4 isoenzyme, patients receiving them are at an increased risk of drug–drug interactions. Considering that imatinib is the most used tyrosine kinase inhibitor, chloroquine may increase imatinib exposure through inhibition of P-glycoprotein, and it may also cause decreased intracellular exposure of imatinib by inhibiting hOCT1 which can lead to therapy failure. In addition to that, imatinib, which is a competitive inhibitor of CYP2D6, can increase the serum levels of chloroquine when co-administered [15].

Like tyrosine kinase inhibitors, chloroquine and azithromycin can also cause QTc prolongation, so close monitoring of the QTc interval together with measurement of electrolytes is crucial in patients with chronic myeloid leukemia and COVID-19 infection who receive these drugs. Favipiravir is an antiviral drug that has also been used in the treatment of patients with COVID-19 infection. This drug has likewise been shown to be associated with QTc prolongation, so caution is required when used in patients with chronic myeloid leukemia who are on tyrosine kinase inhibitor therapy and have COVID-19 infection [15,23].

Previous studies of patients with chronic-phase chronic myeloid leukemia and COVID-19 infection do not indicate that the treatment with TKIs should be delayed or held [23]. It is recommended that patients with chronic myeloid leukemia should continue to receive tyrosine kinase inhibitor during COVID-19 infection, but drug interactions must be considered knowing the fact that both tyrosine kinase inhibitor and drugs used to treat SARS-CoV-2 infection can cause prolongation of the QTc interval. Therefore, regular ECG monitoring with rapid and timely dose modification of these drugs is required [15].

## 5. Conclusions

An analysis of the clinical outcome of COVID-19 infection in patients with chronic myeloid leukemia showed a mild course of the disease with recovery, which is consistent with most previous studies. The use of tyrosine kinase inhibitor therapy in COVID-19-positive patients did not worsen the outcome of the infection but may have played a protective role. It is necessary to analyze a larger number of patients through disease registries in order to confirm or refute these results as well as to develop evidence-based guidelines for the treatment of patients with chronic myeloid leukemia and infection.

## Figures and Tables

**Table 1:** Basic characteristics of COVID-19-positive patients with chronic myeloid leukemia.

Patient Characteristics	Total of 30 Patients
Gender	
Male	13 (43.3%)
Female	17 (56.7%)
Age	
≤65 years	19 (63.3%)
>65 years	11 (36.7%)
Comorbidities	
No comorbidities	9 (30%)
One comorbidity present	8 (26.7%)
Two or more comorbidities present	13 (43.3%)
Last used TKI	
Imatinib	26 (86.7%)
Nilotinib	4 (13.3%)
The last level of therapeutic response	
Molecular response (MR3–MR4.5)	22 (73.4%)
Complete cytogenetic response	6 (20%)
Complete hematological response	1 (3.3%)
No therapeutic response	1 (3.3%)

**Table 2:** Severity of infection, treatment, and outcome of COVID-19-positive patients with chronic myeloid leukemia.

	Total of 30 Patients
Severity of COVID-19 infection according to WHO	
Asymptomatic/Mild	17 (56.7%)
Moderate	7 (23.3%)
Severe/Critical	6 (20%)
TKI therapy during COVID-19 infection	
Interrupted	16 (53.3%)
Continued	14 (46.7%)
Specific therapy for COVID-19 infection	
Antiviral	8 (26.7%)
Antibiotic	27 (90%)
Anticoagulant therapy	13 (43.3%)
Oxygen therapy	4 (13.3%)
Outcome of COVID-19 infection	
Recovery	30 (100%)
Fatal outcome	0 (0%)

## Data Availability

All data underlining this article are available in this article.
